# Identification and biological evaluation of benzimidazole-based compounds as novel TGFβR1 inhibitors

**DOI:** 10.1080/14756366.2025.2600746

**Published:** 2026-01-21

**Authors:** Hui-Ju Tseng, Yi-Wen Wu, Yan-Ling Chen, Tony Eight Lin, Yu-Ting Fang-Chin, Yueh-Lin Wu, Tzu-Ying Sung, Shih-Chung Yen, Jui-Hua Hsieh, Kai-Cheng Hsu, Shiow-Lin Pan

**Affiliations:** a School of Pharmacy, College of Pharmacy, Kaohsiung Medical University, Kaohsiung, Taiwan; b Graduate Institute of Cancer Biology and Drug Discovery, College of Medical Science and Technology, Taipei Medical University, Taipei, Taiwan; c Ph.D. Program for Cancer Molecular Biology and Drug Discovery, College of Medical Science and Technology, Taipei Medical University, Taipei, Taiwan; d Division of Nephrology, Department of Internal Medicine, School of Medicine, College of Medicine, Taipei Medical University, Taipei, Taiwan; e TMU Research Center of Urology and Kidney, Taipei Medical University, Taipei, Taiwan; f Division of Nephrology, Department of Internal Medicine, Wan Fang Hospital, Taipei Medical University, Taipei, Taiwan; g Warshel Institute for Computational Biology, School of Medicine, The Chinese University of Hong Kong (Shenzhen), Shenzhen, Guangdong, China; h Division of Translational Toxicology, National Institute of Environmental Health Sciences, National Institutes of Health, Durham, NC, USA; i TMU Research Center of Cancer Translational Medicine, Taipei Medical University, Taipei, Taiwan; j Cancer Center, Wan Fang Hospital, Taipei Medical University, Taipei, Taiwan

**Keywords:** TGF-β, TGFβR1, structure-based virtual screening, kinase selectivity

## Abstract

TGF-β promotes progression and metastasis in later stages of tumour development, and inhibitors targeting TGF-β or its receptor have faced clinical limitations due to toxicity and poor selectivity. This study aimed to identify novel TGFβR1 inhibitors by screening the ChemDiv database using a structure-based virtual screening approach. Among the top-ranked compounds, 3282–0487 showed the highest potency. Its analogues were further evaluated, leading to four potent TGFβR1 inhibitors with sub-micromolar IC_50_ values. Molecular docking confirmed favourable binding interactions, and structure–activity relationship analysis highlighted key structural features contributing to inhibitory activity. Among these, compound 3282-0486 demonstrated the lowest IC_50_ values against colorectal cancer cells, inducing apoptosis and dose-dependent anti-migration effects. Its efficacy was further supported by changes in downstream TGFβR1 signalling, including p-Smad2, EMT markers, and PARP1 cleavage. Additionally, compound 3282-0486 exhibited selectivity for TGFβR1. Overall, these findings support compound 3282-0486 as a promising TGFβR1 inhibitor with therapeutic potential.

## GRAPHICAL ABSTRACT

**Figure:**
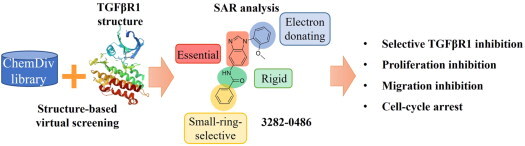

## Introduction

Transforming growth factor-β (TGF-β) is a key cytokine that acts as a multifunctional regulator involved in various biological processes and numerous human diseases, such as cardiovascular disease, fibrosis, and cancer^1^. TGF-β receptors are expressed on the cell surface, including type I (TGFβR1) and type II (TGFβR2) serine/threonine kinase receptors involved in signal transduction. Upon TGF-β binding to TGFβR2, TGFβR1 is phosphorylated at its glycine/serine-rich domain, forming an activated ligand-receptor complex^2^. This complex subsequently phosphorylates Smad2 and Smad3 at their C-terminal serine residues. The phosphorylated Smad2/3 then binds to Smad4 to translocate into the nucleus and regulate gene transcription with additional transcription regulators as co-activators or co-repressors^2^. The intricate signalling mechanisms of TGF-β not only regulate diverse biological processes, but also contribute significantly to the pathogenesis of various diseases, making it a crucial target for therapeutic interventions.

In tumour development, TGF-β is a double-edged sword as TGF-β acts as a tumour suppressor and a tumour promoter in different stages of tumour progression. As a tumour suppressor, TGF-β inhibits cell proliferation, induces apoptosis, and regulates autophagy. TGF-β can induce cell cycle arrest at the G1 phase through the downregulation of MYC and G1 cyclin-dependent kinases (CDKs). However, as a tumour promoter, TGF-β and TGFβR1 promote tumour invasion, metastasis, tumour stem cells, and immune evasion during tumour development^3^^,^^4^. During tumour development, some cancers evade TGF-β-induced G1 arrest and instead utilise TGF-β to promote tumour progression. The TGFβR1 and SMAD signalling are known to be involved in epithelial-to-mesenchymal transition (EMT), which is a marker of tumour progression, invasion, and metastasis^5^^,^^6^. TGF-β also activates phosphoinositide 3 kinase (PI3K), AKT, and mTOR^7^. Increased expression of TGF-β in serum levels and its receptor has been identified in various cancers during the late stages of tumour progression, such as breast cancer, colorectal cancer, and prostate cancer^8^^,^^9^. Overexpression of TGF-β and TGFβR1 is associated with angiogenesis, metastasis, and poor prognosis^10^. TGF-β also acts as an immunosuppressive cytokine by inhibiting the development, proliferation, and activation of CD4, CD8 T cells, NK cells, and macrophages. Further, higher plasma levels of TGF-β have been associated with increased resistance to chemotherapy^11^. These findings indicate that TGF-β and TGFβR1 play an important role in tumour progression, invasion, and metastasis and suggest that TGF-β and TGFβR1 are potential targets in regulating tumour progression.

TGFβR1 has been considered a promising therapeutic target due to its critical role in tumour progression. To date, several TGFβR1 inhibitors have entered clinical or preclinical studies, such as Galunisertib and SB-431542^12^. Galunisertib, when combined with 5-FU-based chemotherapy, improved treatment outcomes in patients with locally advanced rectal cancer^13^. However, it has demonstrated cardiac toxicity in preclinical studies^14^^,^^15^. SB-431542 inhibited the proliferation and migration of glioma cells and other solid tumours^16^^,^^17^. Despite this, it lacked sufficient selectivity and potency for clinical application^18^. As a result, no further clinical trials with these inhibitors are currently ongoing^19^. In addition, several other TGFβR1 inhibitors have shown promise in preclinical studies. However, their performance in clinical applications remains modest^15^. The main limitations of current inhibitors may include toxicity and poor selectivity. Therefore, the development of a new generation of small molecules targeting TGFβR1 is essential to improve therapeutic outcomes.

**Figure 1.**
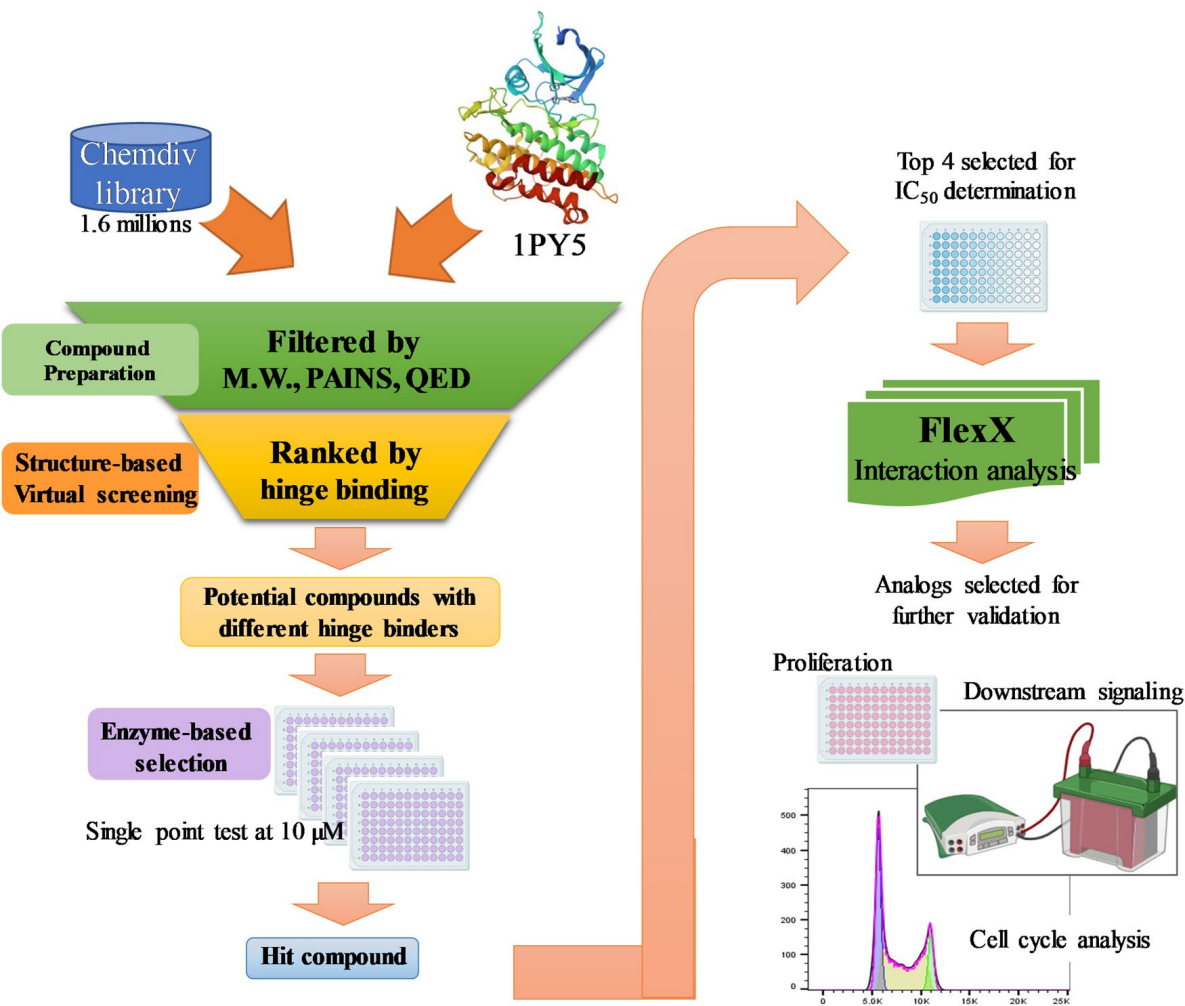
Schematic workflow for identifying TGFβR1 inhibitors. The compound library was prepared to minimise false positives and eliminate compounds with poor drug-like properties. Subsequently, molecular docking was performed. The docking scores and interactions with hinge residues were utilised to identify potential compounds. Then, the compounds were tested for TGFβR1 inhibitory activity. The structure and activity relationship was further evaluated, and the interaction analysis was performed. The validated compounds were subsequently selected for evaluation in cell-based assays.

In this study, we aimed to identify new TGFβR1 inhibitors with novel scaffolds using structure-based virtual screening (SBVS), followed by enzyme-based and cell-based assays (Figure 1). Compared with traditional high-throughput screening, SBVS shows advantages in enhancing efficiency and cost-effectiveness^20^^,^^21^. We have previously applied this strategy to identify new small-molecule inhibitors targeting kinases^22^^,^^23^. In this work, compounds from ChemDiv were docked into the binding site of TGFβR1, and the top-ranked compounds were selected based on docking scores and key interactions. These compounds were subsequently evaluated using a kinase activity assay. Compounds with validated activity were further evaluated for their cytotoxicity, anti-migration effects, and cell-cycle effects in colorectal cancer cells. These findings led to the identification of novel TGFβR1 inhibitors with potential therapeutic applications in colorectal cancer.

## Materials and methods

### Preparation of compound library

The compound screening library was obtained from the ChemDiv database (www.chemdiv.com) and consisted of approximately 1.6 million compounds. The library was prepared using BIOVIA Pipeline Pilot^24^. This process included the removal of compounds with molecular weights greater than 1000 Da and those containing pan-assay interference compounds (PAINS).^25^ PAINS substructures were sourced from an online repository (https://www.zbh.uni-hamburg.de/forschung/amd/datasets/smarts-dataset.html). A high-throughput screening filter was applied to eliminate compounds containing non-organic atom types or reactive substructures. In addition, drug-likeness was assessed using the quantitative estimate of drug-likeness (QED) component, and compounds with QED scores below 0.4 were excluded.^26^

### Molecular docking

The compounds from the screening library were docked into the binding site of TGFβR1. The crystal structure TGFβR1 (PDB ID: 1PY5) was obtained from the Protein Data Bank. The docking protocol was performed using the BioSolveIT nodes in the KNIME suite (https://www.biosolveit.de/knime/). The protein structure was prepared by removing any water molecules, and the binding site was defined based on the location of the co-crystallized ligand (Ligand ID: PY1). The three-dimensional conformations of the compounds for docking were generated using the 3D Coordinates Generator node. For docking, FlexX program was used with default settings^27^. The docked compounds were then ranked according to their docking scores. The top-ranked compounds were selected for protein-ligand interaction analysis using BIOVIA Pipeline Pilot^24^. Compounds lacking hydrogen bonding interactions with the hinge residue of TGFβR1 were excluded. Finally, the top-ranked molecules were visually inspected, and available potential inhibitors were selected for testing.

### Compound origin and specifications

All screening compounds used in this study were obtained from ChemDiv (San Diego, CA, USA; https://www.chemdiv.com/catalogue/screening-libraries/). According to the specifications provided by ChemDiv, each compound was subjected to critical quality control testing, including liquid chromatography-mass spectrometry (LC-MS) and nuclear magnetic resonance (NMR) analyses to ensure a purity threshold greater than 90%. Compounds were supplied in dry powder form to maintain stability. Catalogue numbers reported in this manuscript correspond to identifiers assigned by ChemDiv.

### Kinase inhibitory assay

The kinase inhibitory assay was performed using the SelectScreen Kinase Profiling Service from Thermo Fisher Scientific (www.thermofisher.com/selectscreen). Specifically, the LanthaScreen Eu binding assay was used to evaluate the inhibitory activity of compounds against TGFβR1. In this assay, the kinase, test compound, fluorescent tracer, and europium (Eu)-labelled antibody were mixed in solution. The tracer binding to the target kinase was detected by the Eu-labelled antibody. When both the tracer and antibody were bound to the kinase, a high fluorescence resonance energy transfer (FRET) signal was generated. In contrast, disruption of this binding by a TGFβR1 inhibitor resulted in a reduced FRET signal. Each enzymatic activity assay was conducted and monitored in accordance with the quality control standards provided by Thermo Fisher Scientific. In addition, compound selectivity was determined using the Eurofins kinase profiling service.

### Cell culture

HT29 (HTB-38) and HCT116 (CCL-247) were obtained from American Type Culture Collection (ATCC, Manassas, VA, USA). HT29 cells were cultured in Dulbecco’s Modified Eagle Medium (DMEM, Gibco, ThermoFisher Scientific). HCT116 cells were cultured in McCoy’s 5 A modified medium (Merck). The medium was supplied with 10% foetal bovine serum (FBS, ThermoFisher Scientific) and 1% penicillin, streptomycin, and Amphotericin B (Antibiotic-Antimycotic, Gibco, ThermoFisher Scientific). All cells were maintained at 37 °C, 5% CO_2_ in a humidified atmosphere.

### Cytotoxicity analysis

Cells were seeded in 96-well plates at a density of $3\times 10^{3}$ cell per well. After 24 h, cells were incubated with medium containing compounds at various concentrations for 48 h. After incubation, the compound-containing medium was removed, and the cells were treated with medium containing 3-(4,5-dimethyl-2-thiazolyl)-2,5-diphenyl-2H-tetrazolium bromide (MTT, Sigma Aldrich) at a concentration of 0.5 mg/mL for 4 h. After incubation, the medium was removed, and the MTT formazan was dissolved with DMSO. The absorbance was obtained at 590 nm. The data were plotted based on a sigmoidal dose-response equation, and the IC_50_ values were calculated using Graphpad Prism 9.5.1 software (San Diego, CA, USA). The experiments were performed in triplicate.

### Cell cycle analysis

Cells were seeded in 6-well plates at a density of $5\times 10^{4}$ cells per well. After 24 h, cells were incubated with medium containing compounds at various concentrations for 24 h. After incubation, cells were trypsinized and collected in tubes. Cells were fixed with ice-cold ethanol for 30 min, then washed with phosphate-buffered saline (PBS), treated with RNase A (100 μg/mL, ThermoFisher Scientific), and stained with propidium iodide (50 μg/mL, Sigma Aldrich). DNA content of cells was analysed using BD FSCLyric and FlowJo software.

### Scratch assay

Cells were seeded in 24-well plates at a density of $1\times 10^{5}$ cell per well. After 24 h, cells reached 80% confluence, and each well was scraped using a 1 mm pipette tip to create a straight line, referred to as the wound. After scratching, cells were gently washed using fresh medium to remove the detached cells. Images were taken using a microscope (SDPTOP XDF) and a 12.5MP microscope camera (Mshot MSX2-C). After imaging, the medium was replaced with the compound-containing medium and incubated for another 24 h. After 24 h, images were taken again. The distance of the wound was measured, and the percentage of wound closure was calculated.

### Western blotting

Cells were seeded in 6-well plates at a density of $5\times 10^{5}$ cells per well. After 24 h, the medium was replaced with 10 ng/mL TGF-β (#100-21-10UG, Gibco) and compound-containing medium with various concentrations for 24 h. After incubation, cells were trypsinized, washed with DPBS, and lysed with lysis buffer (20 mM Tris, 100 mM MgCl_2_, 125 mM NaCl, 1% Triton X-100) and protease inhibitor cocktail (cOmplete^TM^, Roche). The proteins were separated by 10-15% SDS-PAGE and transferred onto PVDF membranes. The membranes were blocked with 5% skim milk. The proteins were probed with antibodies: anti-GAPDH mAb (#60004-2-Ig, Proteintech), anti-E-cadherin pAb (#20874-1-AP, Proteintech), anti-Vimentin pAb (#10366-1-AP, Proteintech), anti-phospho-SMAD2 (Ser465, Ser467) (#44-244 G, Invitrogen), and anti-PARP1 mAb (#MA5-15031, Invitrogen). The primary antibodies were recognised with secondary antibodies conjugated with horseradish peroxidase (HRP): goat anti-mouse IgG (#A28177, Invitrogen) and goat anti-rabbit IgG (#A27036, Invitrogen). The signals were detected using Chemidoc MP (Bio-Rad) with enhanced chemiluminescent substrate (ECL, SuperSignal^TW^, West Pico PLUS, ThermoFisher Scientific).

### Statistical analysis

All statistical analyses were performed and visualised using Graphpad Prism. Concentration-response curves were fitted using a three-parameter model (nonlinear regression), and IC_50_ values were calculated accordingly. Immunoblot data were quantified with ImageJ software (National Institutes of Health Bethesda, MD, USA). Statistical results were presented as the mean ± standard deviation from at least three independent experiments. A *p* values of <0.05 was considered statistically significant, denoted as * *p* < 0.05, ** *p* < 0.01, *** *p* < 0.001.

## Results

### Virtual screening identifies potential inhibitors

We identified potential TGFβR1 inhibitors using SBVS. The screening was conducted using FlexX^27^.Compounds from the screening library were docked into the binding site of TGFβR1 using the crystal structure (PDB ID: 1PY5) and ranked based on their docking scores. Typical kinase inhibitors targeting the ATP binding site form hydrogen bonds with residues in the hinge region of the kinase^28^. Therefore, compounds lacking hydrogen bonding interactions with hinge residues D281, L282, or H283 were excluded. The top 400 ranked compounds that met these criteria were further analysed for favourable spatial orientation within the TGFβR1 binding site. Finally, selected molecules were checked for availability. A total of 15 compounds, each containing a different hinge binding scaffold, were selected for testing.

The selected compounds were tested using a kinase inhibitory assay at a concentration of 10 μM (Table 1). Among these, two compounds, 3282–0487 and 8016-6700, exhibited greater than 50% inhibition of TGFβR1 activity, indicating promising inhibitory potential. In particular, compound 3282-0487 showed the highest inhibition at 96%. Based on this result, we searched for analogs of 3282-0487 and tested 16 related compounds. Among these analogs, five compounds demonstrated greater than 50% inhibition (Table 2). Compounds producing inhibition greater than 70% were selected for further testing to determine their IC_50_ values. Compound 7491-0241 showed the most potent activity with an IC_50_ value of 313.6 nM, followed by 3282–0486 (363.1 nM), 3282-0487 (379.1 nM), and 7491-0127 (391.7 nM) (Table 3). These results suggest that a potent TGFβR1 inhibitor was identified.

**Table 1.** Inhibitory activity of selected compounds against TGFβR1 at 10 μM.

Compound	% Inhibition (10 μM)	Compound	% Inhibition (10 μM)
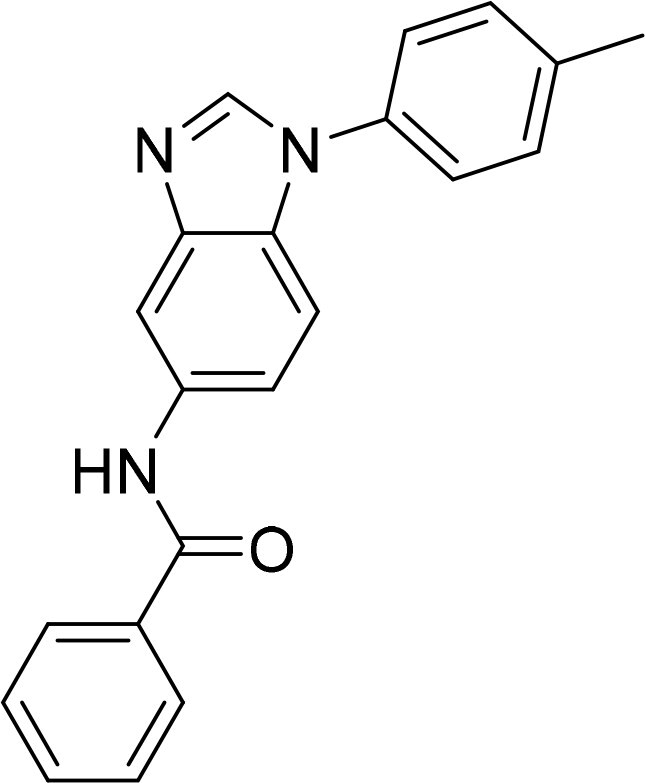 3282-0487	96	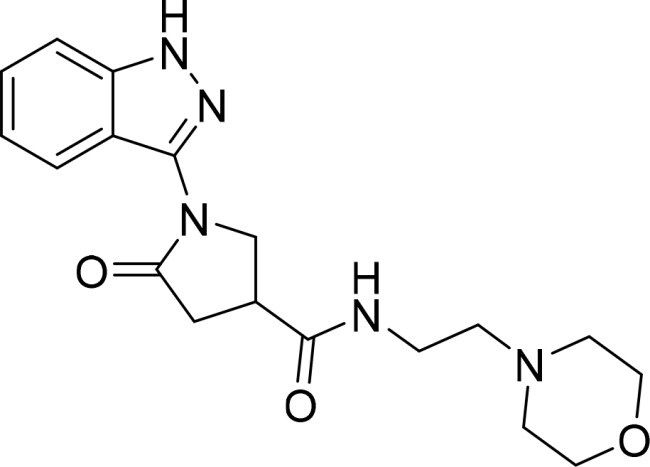 J106-0204	32
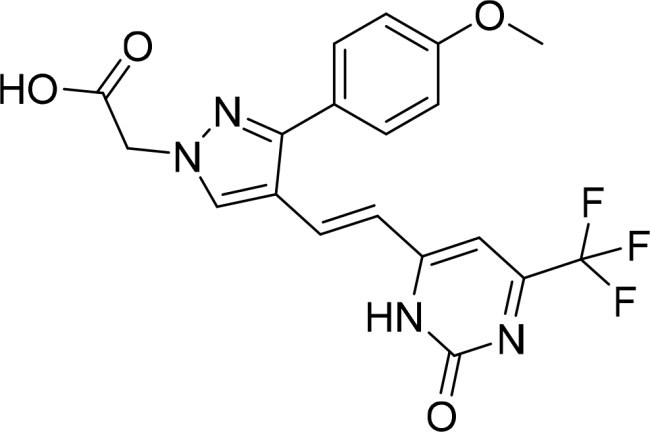 8016-6700	55	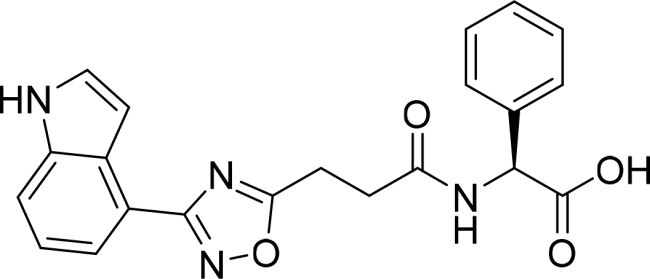 Y043-5133	29
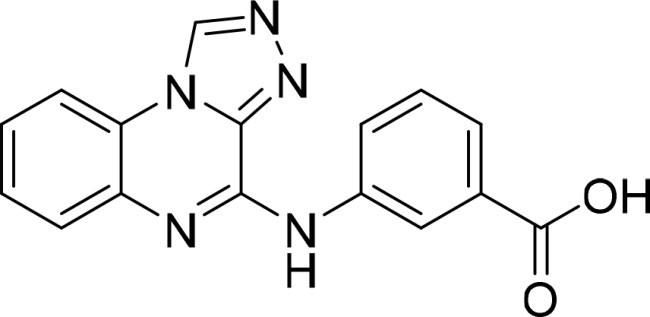 C735-0109	44	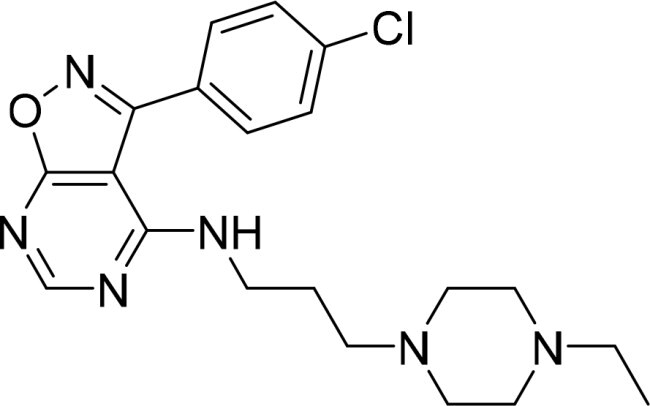 C660-0735	17
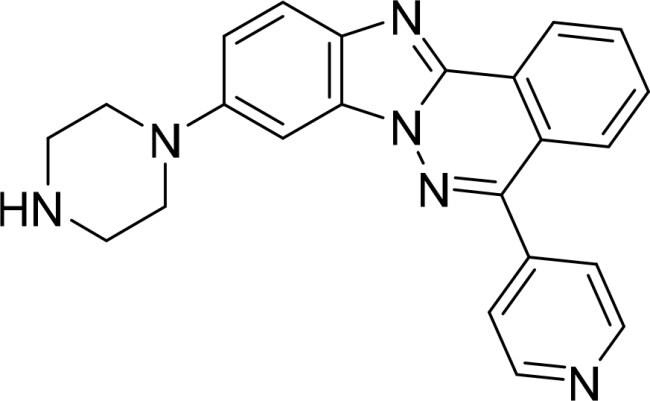 ZC14-0020	39	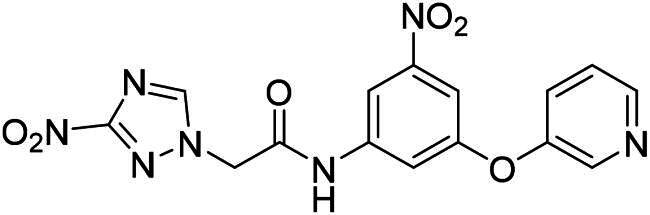 1762-0139	17
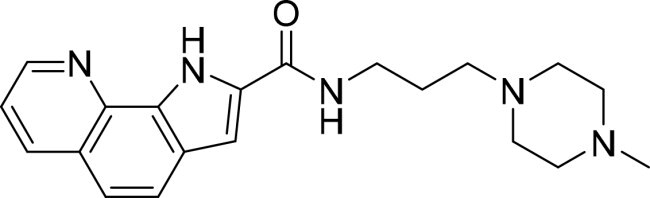 E950-0094	37	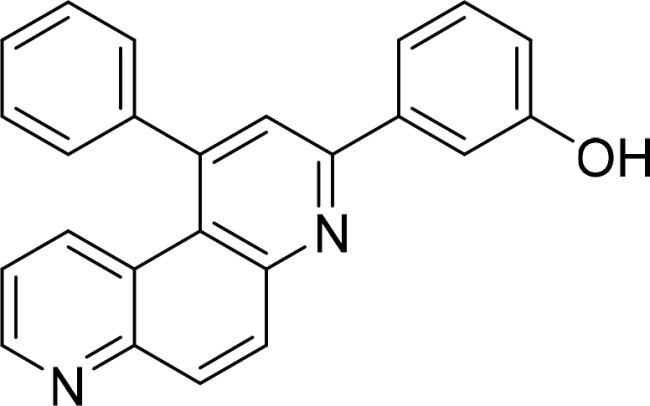 8011-4043	15
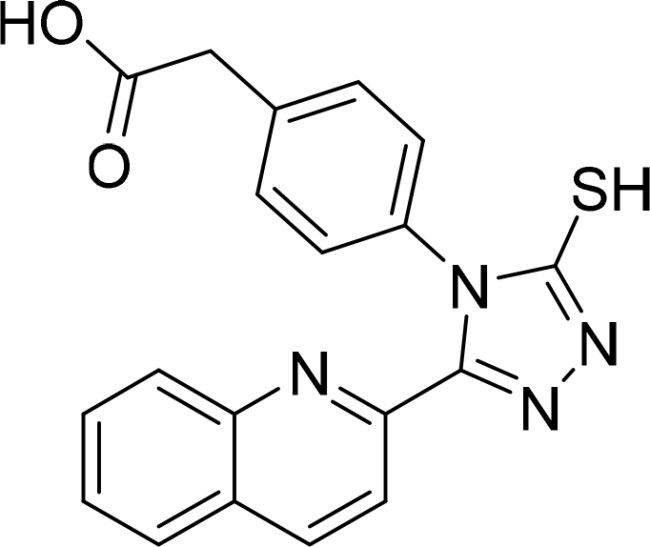 7832-0107	36	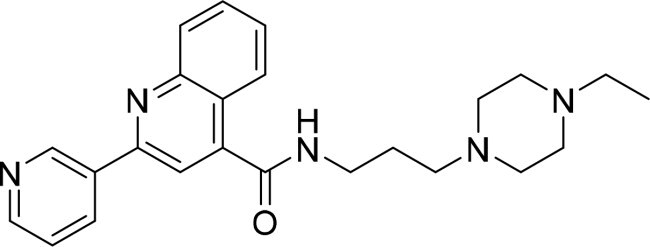 G547-0046	12
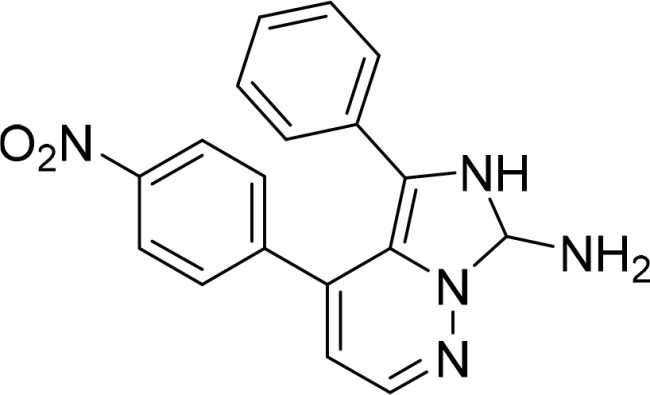 Y021-2226	36	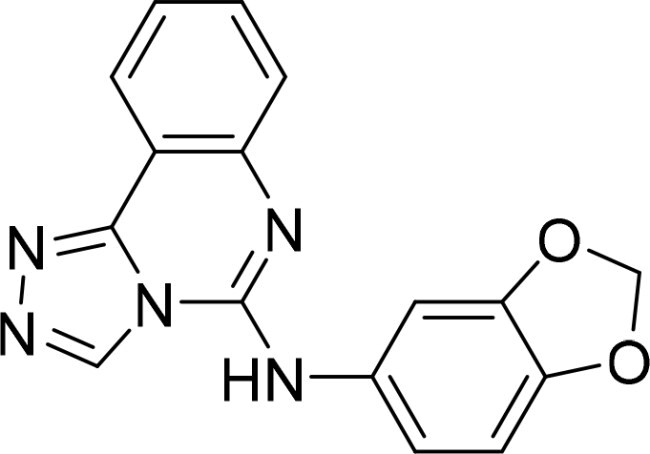 E881-0008	0
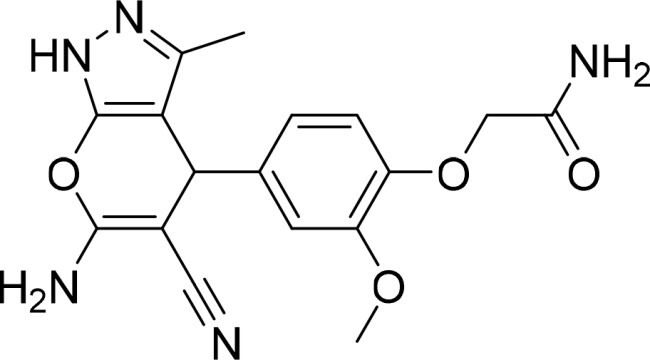 4303-0785	32		

**Table 2.** Inhibitory activity of compound 3282-0487 and its analogues against TGFβR1 at 1 μM.

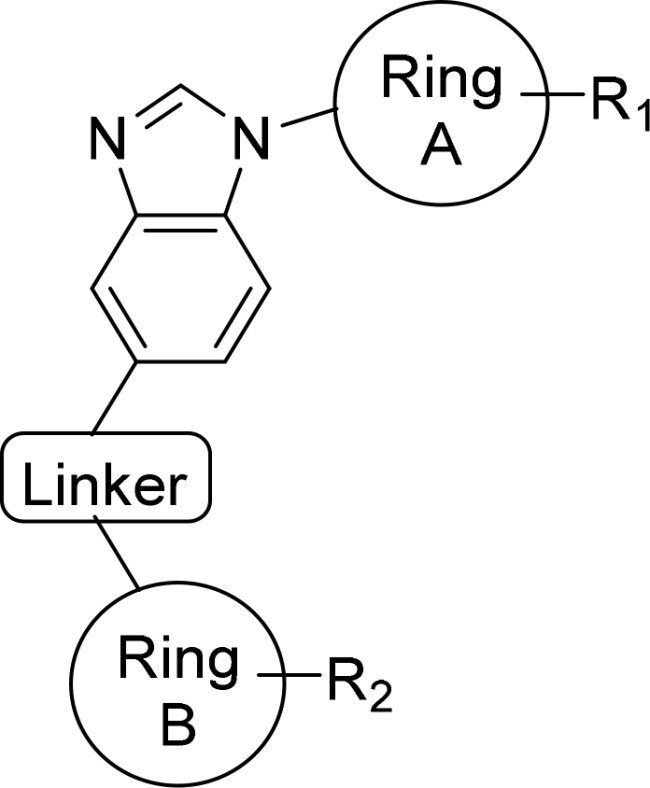									
Compound	Ring A/R1	Linker	Ring B/ R2	% Inhibition (1 μM)	Compound	Ring A/R1	Linker	Ring B/ R2	% Inhibition (1 μM)
3282-0487	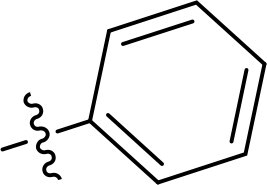	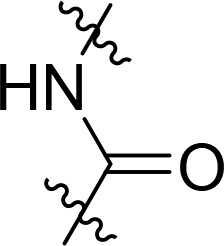	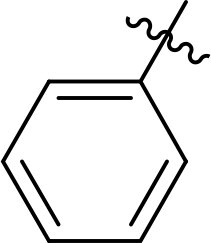	73	1773–0132	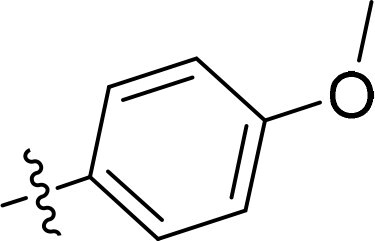	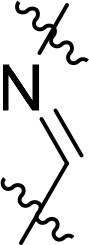	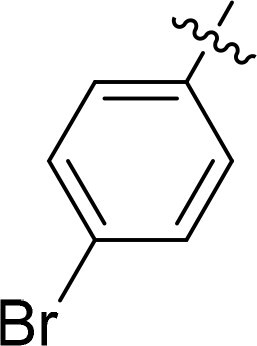	15
3282–0486	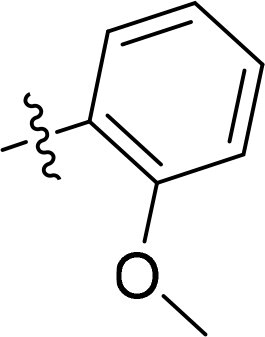	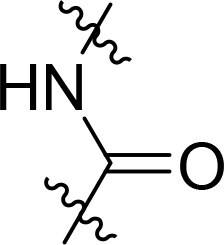	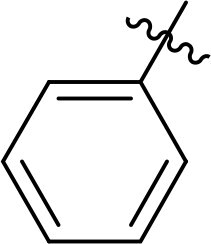	74	3282–0451	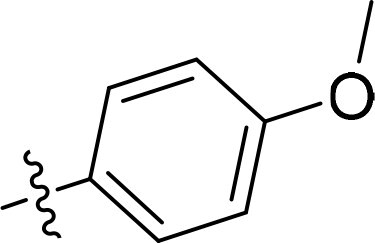	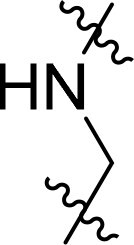	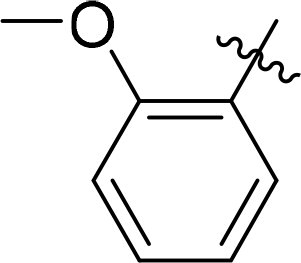	10
3546–0618	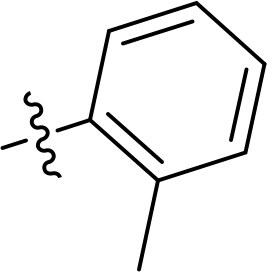	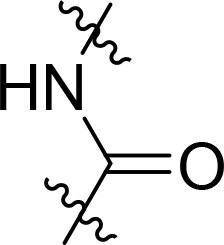	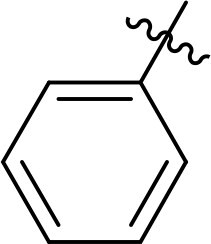	51	7491–0127	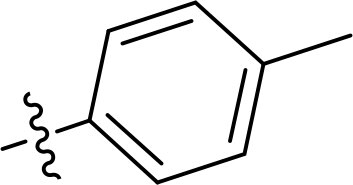	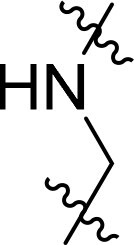	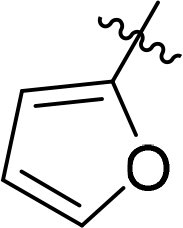	75
3546–0634	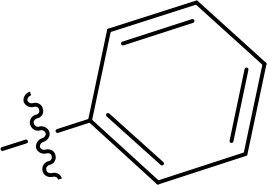	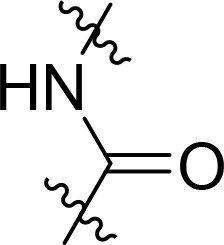	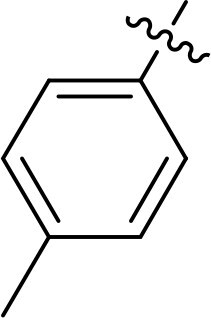	12	K783-0351	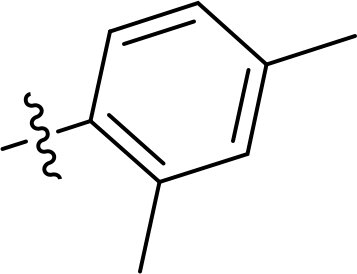	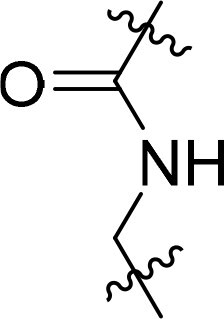	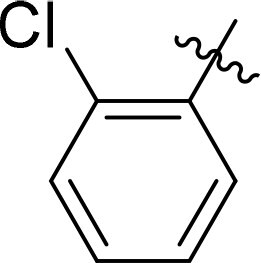	12
7491–0241	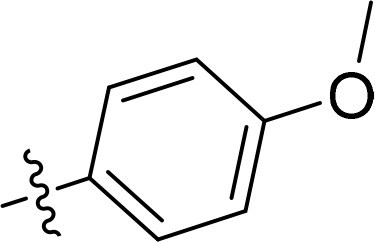	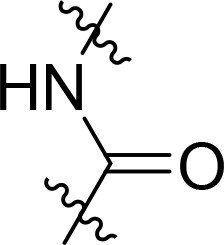	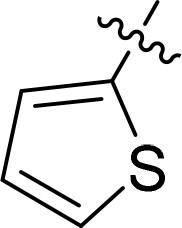	76	K783-0334	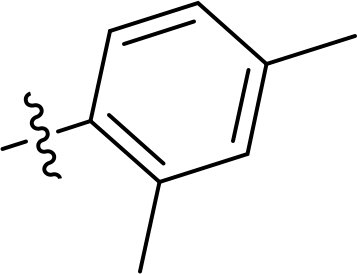	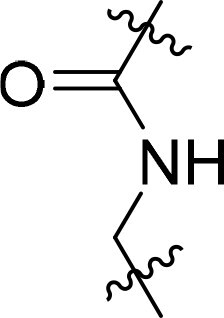	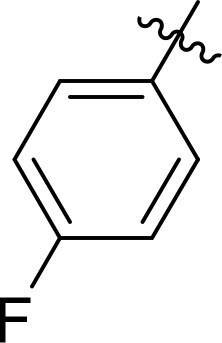	7
K783-0337	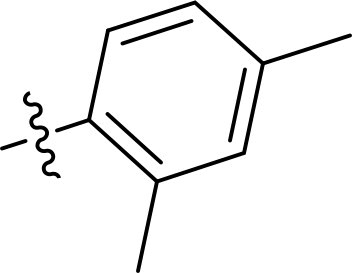	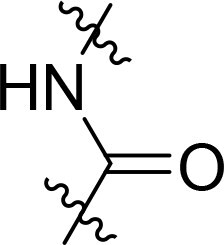	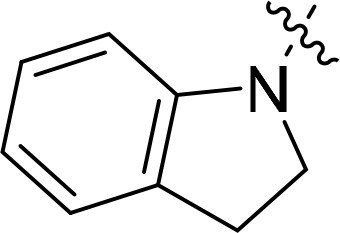	11	K783-0570	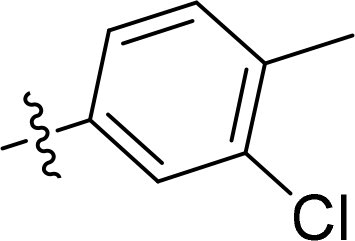	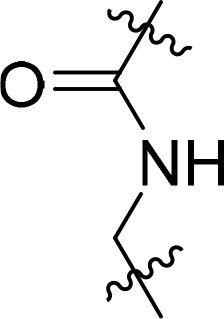	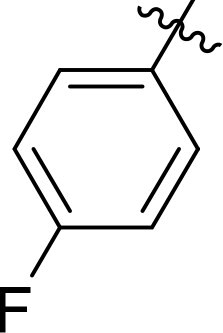	19
5326–0669	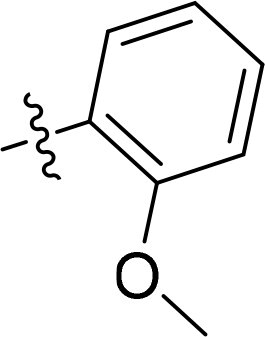	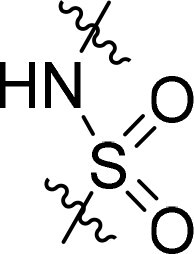	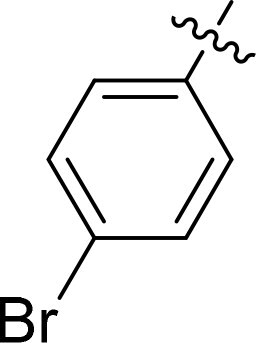	14					

**Table 3.** IC_50_ values of four identified compounds.

Compound	IC_50_ (nM)
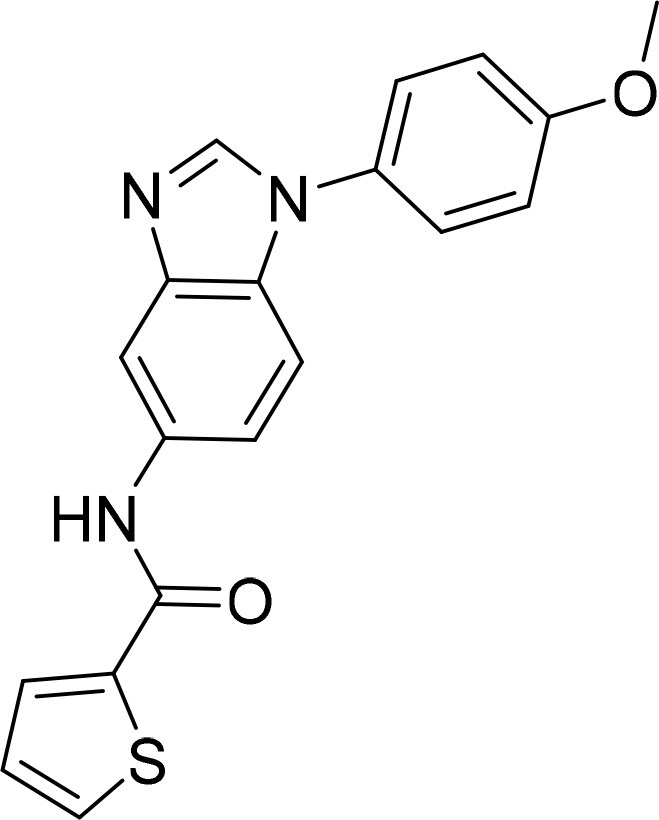 7491–0241	313.6
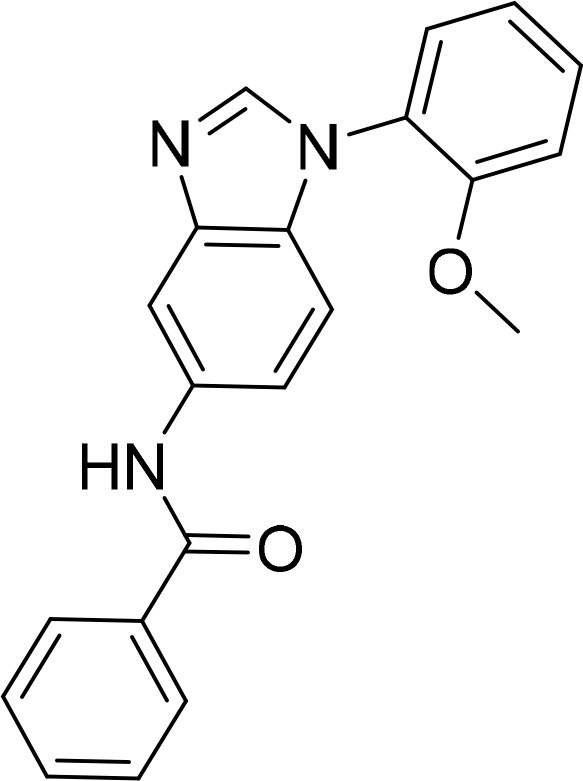 3282–0486	363.1
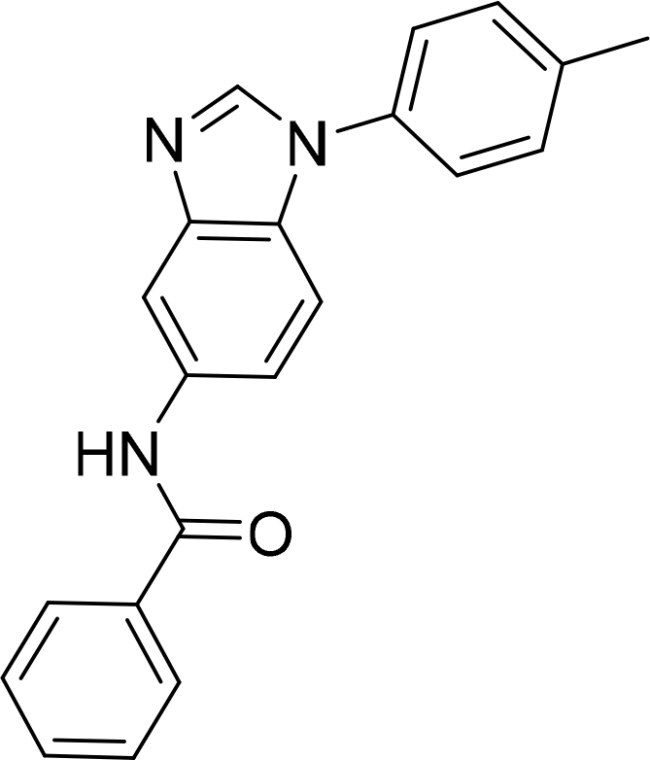 3282–0487	379.1
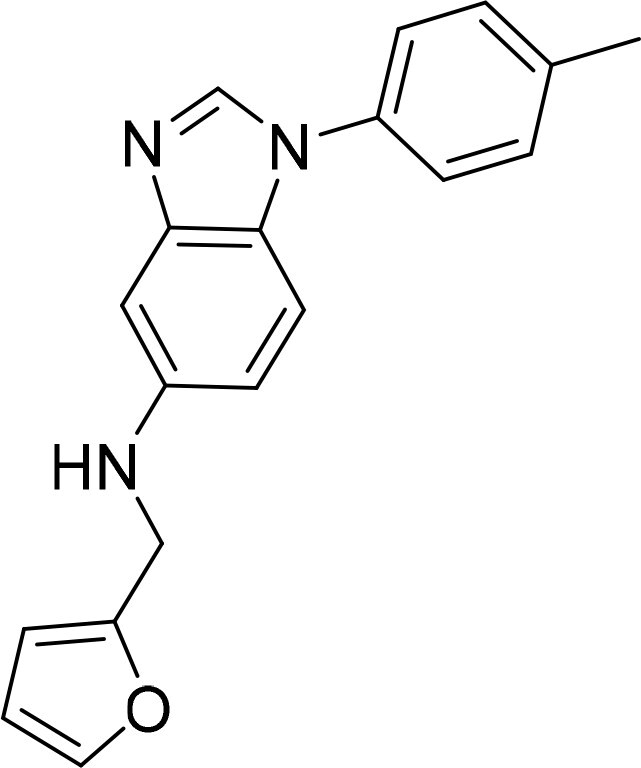 7491–0127	391.7

### Interaction analysis of identified inhibitors

**Figure 2.**
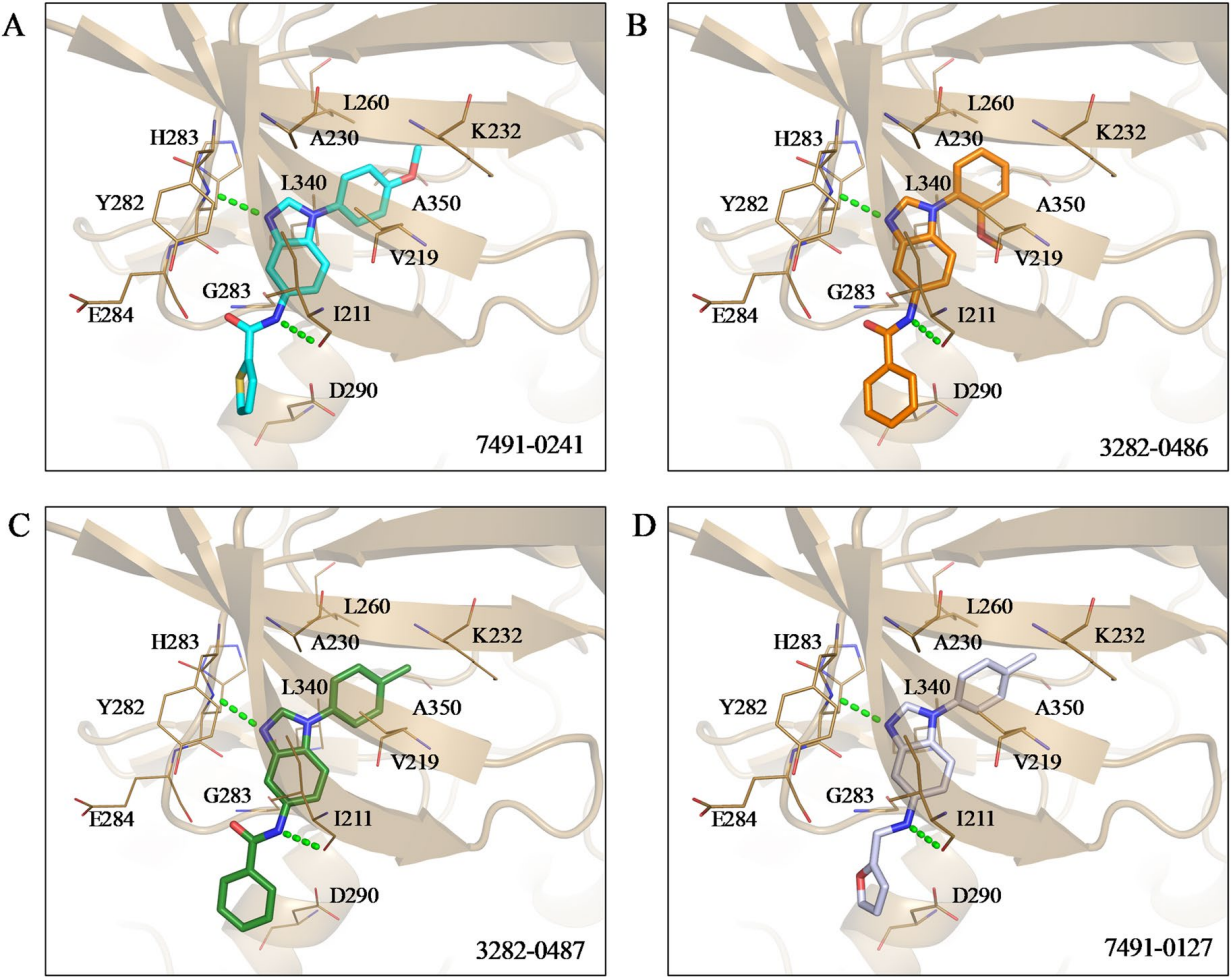
Docking poses of identified inhibitors. Analysis of the docking pose for (A) 7491-0241, (B) 3282–0486, (C) 3282-0487, and (D) 7491-0127 shows favourable positioning within the TGFβR1 binding site. This includes a hydrogen bond to the hinge residue H283. The TGFβR1 structure is represented as a cartoon, and the binding site is represented as sticks and labelled where appropriate. Hydrogen bonds are denoted as a dashed green line.

Kinase inhibitors targeting the ATP-binding site have a common binding pattern.^28^ Analysis of the docking poses of the four compounds showed that they share similar protein-ligand interactions (Figure 2). This pattern aligns with the observed potencies (Table 3).A hydrogen bond to the hinge residue H283 was observed. At least one hydrogen bond to the kinase hinge residues is generally required for inhibitors targeting the binding site^28^^,^^29^. This is facilitated by the benzimidazole scaffold shared by the molecules. An additional hydrogen bond was observed for residue I211, which is located on the periphery of the binding site. These interactions serve to “anchor” the benzimidazole scaffold in the binding site. A hydrophobic interaction between residue V219 and the benzimidazole was also observed. Additionally, the benzimidazole scaffold shared across the four molecules is flanked by aromatic rings. This facilitates hydrophobic interactions at the interior of the binding site with residues A230, K232, L260, and L340, and A350. Meanwhile, the ring in the periphery forms hydrophobic interactions with residue I211. These interactions suggest that the molecules create favourable interactions with the TGFβR1 binding site.

### Exploring the structure-activity relationship of compounds

**Figure 3.**
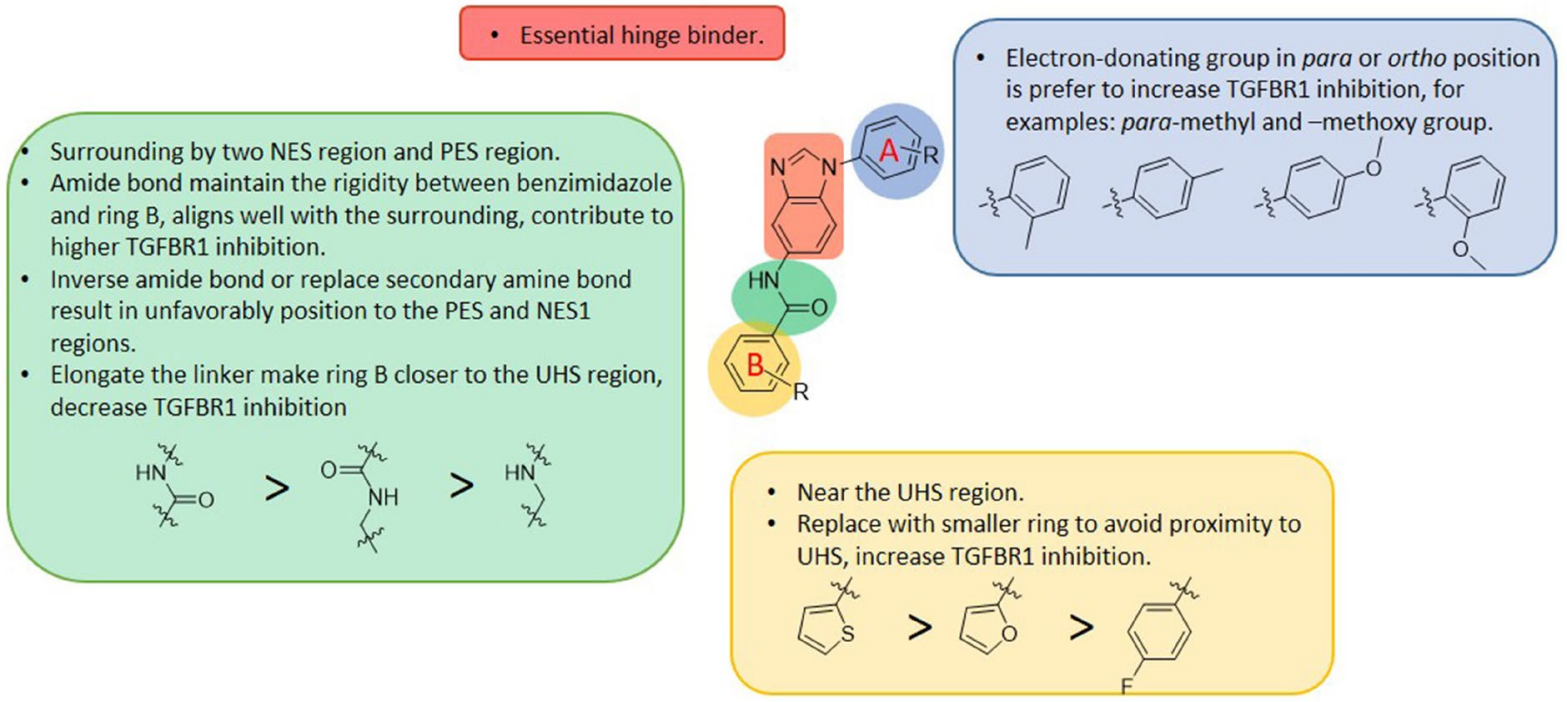
Overview of structure–activity relationship of 3282–0487 and related compounds. The SAR analysis was conducted based on 3282-0487 and 16 tested analogs to generate this SAR map. The essential hinge binder is marked in rose red. Ring A is marked in blue, Ring B is marked in yellow, and the linker connecting the hinge binder and Ring B is marked in green.

The structure-activity relationships (SARs) of 3282-0487 and its analogs were analysed (Figure 3). The benzimidazole group was identified as an essential hinge binder. Substitution of Ring A with electron-donating groups at the ortho or para positions was generally associated with slightly increased TGFβR1 inhibition, as observed in comparing 3282-0487 with 3282–0486 and 7491-0241. Ring B is located near an unfavourable hydrophobic site (UHS) (Supporting information Figure S1–S4). Replacing the phenyl ring with thiophene (*e.g.*, 7491-0241) or furan (*e.g.*, 7491-0127) positions at ring B further from the unfavourable hydrophobic site (UHS) enhances TGFβR1 inhibition. The linker connecting the hinge binder and ring B is surrounded by two negative electrostatic sites (NESs) and one positive electrostatic site (PES) (Figure S1). The amide bond maintains the rigidity between benzimidazole and ring B, resulting in a spatial arrangement of the linker that aligns well with the surrounding PES, NES1, and NES2 regions. However, the orientation of the amide bond plays a critical role. TGFβR1 inhibition was more effective when the amine group was positioned closer to benzimidazole than when it was closer to ring B. Additionally, elongation of the amide linker positions ring B closer to the UHS region, which decreases the TGFβR1 inhibition. Further, the imine and amine linkages were unfavourably positioned relative to the PES and NES1 regions, resulting in the diminished TGFβR1 inhibition. Overall, the SAR analysis of 3282-0486 and related compounds highlighted the critical role of the benzimidazole hinge binder, linker conformation, and preferred moieties in Ring A and Ring B in determining TGFβR1 inhibition.

**Figure 4.**
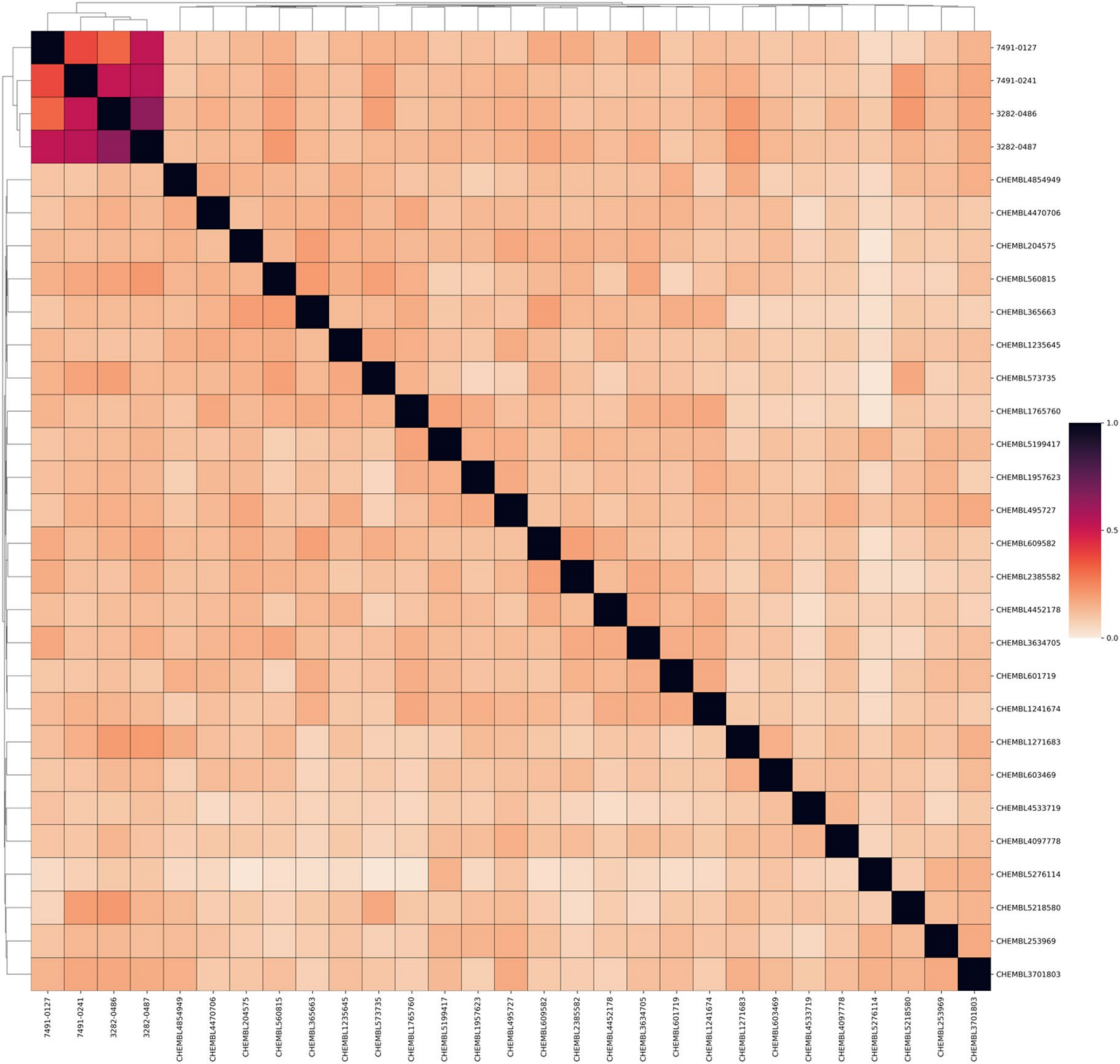
Similarity matrix of identified inhibitors with known TGFβR1 inhibitors. Known TGFβR1inhibitors were obtained from ChEMBL 35. The compounds were clustered, and 25 representative molecules were selected. A Tanimoto similarity score was calculated for each molecule-molecule pair. As structural analogues, the four hit molecules were most similar to each other.

### Structural analysis of TGFβR1 inhibitors

A goal of SBVS is to identify structurally novel inhibitors. To evaluate structural novelty, the identified inhibitors 7491-0241, 3282-0486, 3282-0487, and 7491-0127 were compared to known TGFβR1 inhibitors obtained from ChEMBL 35^30^. The TGFβR1 inhibitors were clustered, and 25 structurally diverse compounds were selected. These known inhibitors were then combined with 3282-0487 and 8016–6700, and a Tanimoto similarity score was generated for each pair^31^. The resulting similarity matrix produced a distinct cluster for the identified inhibitors (Figure 4). This was expected due to their shared structural features. In contrast, the known TGFβR1 inhibitors did not yield a Tanimoto score higher than 0.208 when compared to these two compounds. These findings suggest that the four compounds could serve as novel starting points for the development of potent TGFβR1 inhibitors.

### TGFβR1 inhibitors inhibit colorectal cancer cell proliferation

**Figure 5.**
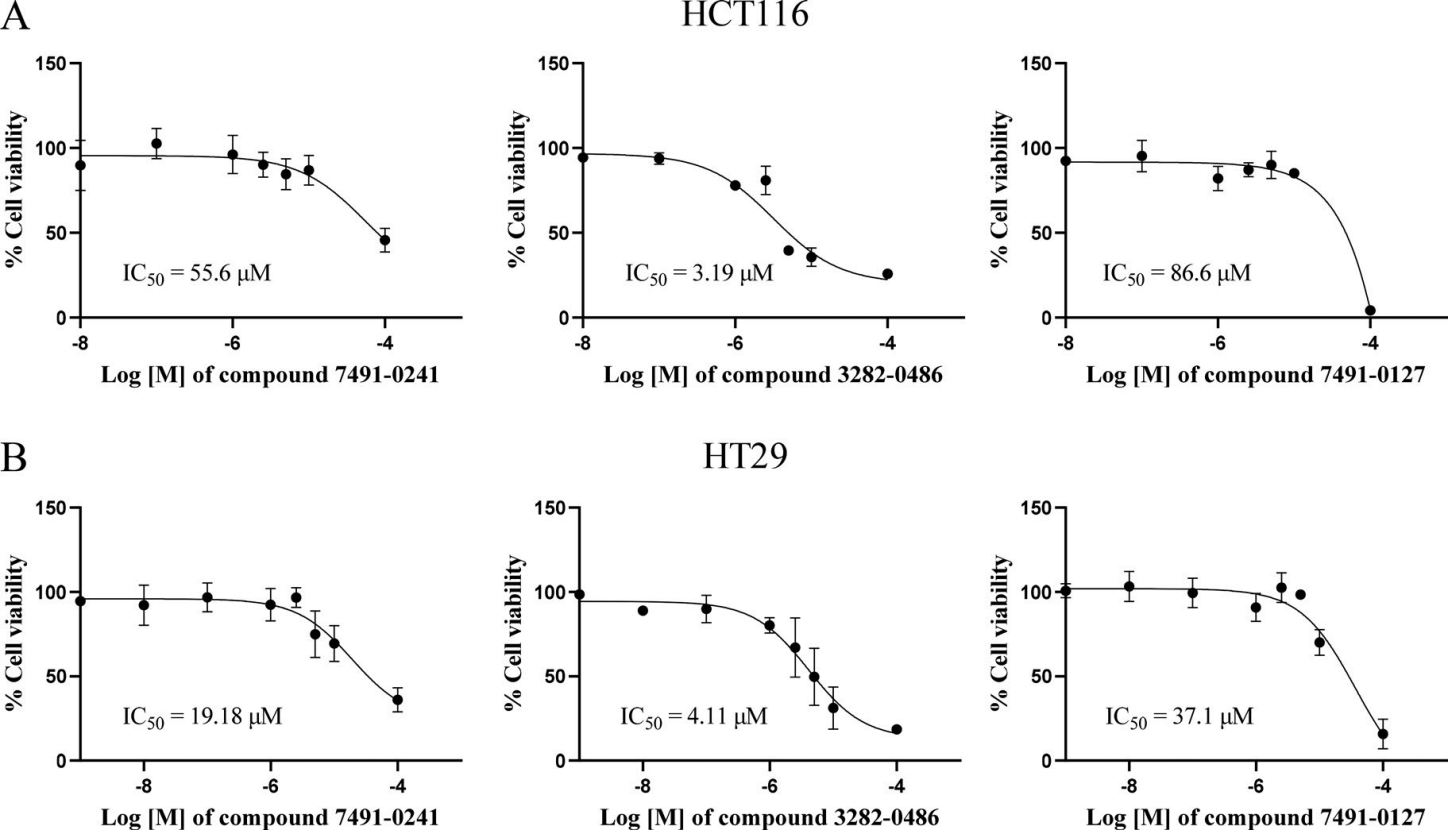
Anti-proliferative activity of TGFβR1 inhibitors against human colorectal cancer cell lines. HCT116 or HT29 cells were treated with the inhibitors at the indicated dose for 48 h. After incubation, the compound-containing medium was removed, and the cell viability was detected with MTT. The experiment was performed in triplicate.

To evaluate the cytotoxic effects of TGFβR1 inhibitors, four compounds with sub-micromolar IC_50_ values were tested in two human colorectal cancer cell lines, HCT116 and HT29. These two cell lines possess different characteristics; the p53 tumour suppressor gene is wild-type in HCT116 cells but mutated in HT29 cells, while the K-Ras oncogene is mutated in HCT116 cells but wild-type in HT29 cells^32^. Both HCT116 and HT29 cell lines have been shown to stably express functional TGFβRs and respond to TGF-β signalling^33^. MTT assay was used to determine the percentage of cell viability of HCT116 and HT29 cells under the treatment of TGFβR1 inhibitors at 10 and 1 µM. All compounds exhibited cell cytotoxicity at 10 µM and reduced cell viability by 12-75% (Table 4). Compound 3282-0486 exhibited the strongest cytotoxic effect at 10 µM. Consequently, the IC_50_ values were further determined at various concentrations (Figure 5). The IC_50_ of compound 3282–0487 was not determined, since its cell viability was above 50% at 100 µM. The effects of compounds 7491-0241 and 7491-0127 in reducing cell proliferation were pronounced in HT29 cells compared to HCT116 cells. Compound 3282-0486 demonstrated the best cytotoxicity against both cell lines (3.19 and 4.11 µM). Therefore, compound 3282-0486 was selected for further evaluation.

**Table 4.** Percentage of cell viability of the inhibitors.

Compound	% Cell viability at 10 µM	% Cell viability at 1 µM
7491-0241	75.1	106.1
3282-0486	24.3	88.2
3282-0487	88.8	101.7
7491-0127	85.3	91.5

### 3282-0486 Induced cell cycle arrest at G2/M phases

**Figure 6.**
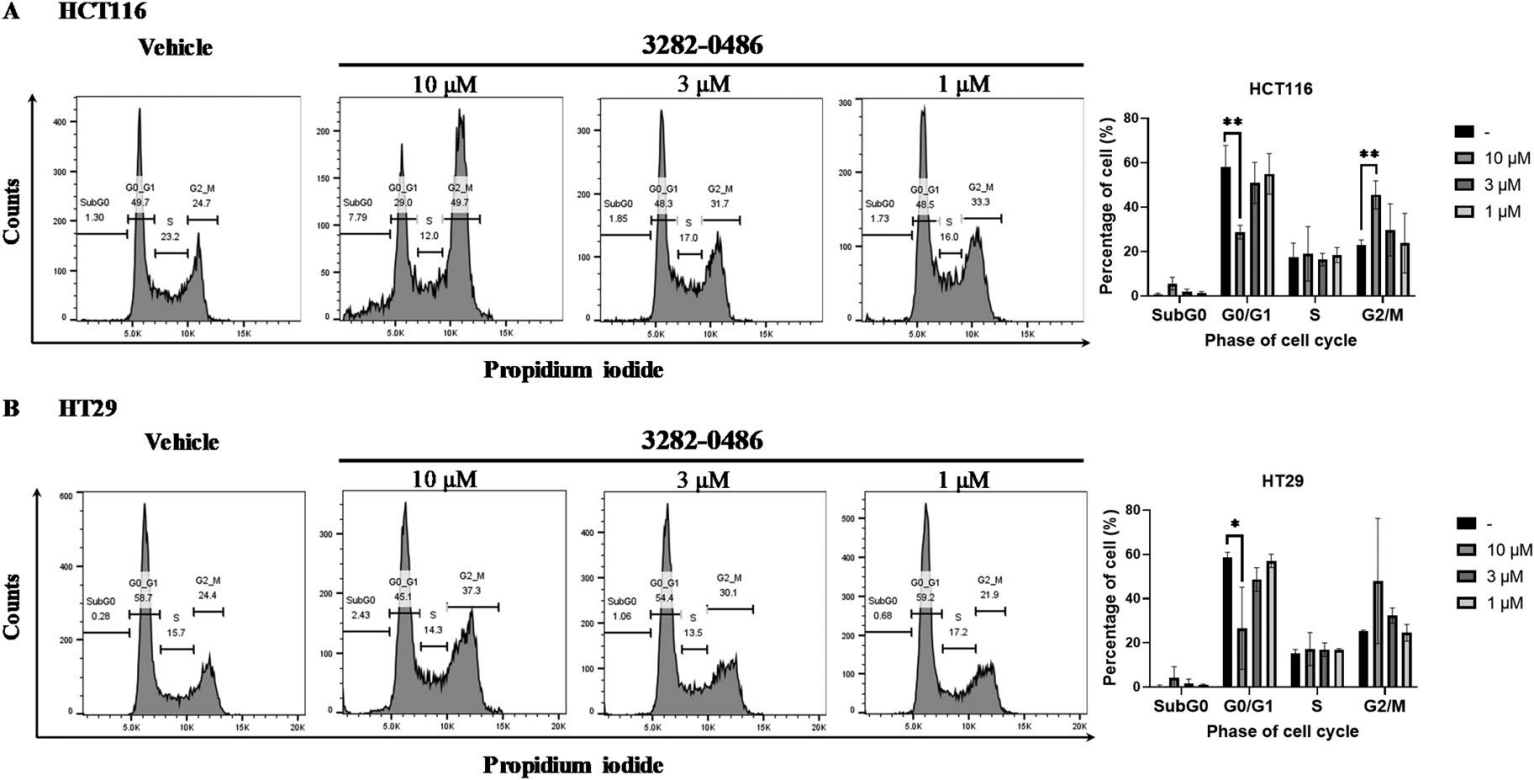
G2/M arrest of compound 3282-0486 in colorectal cancer cells. (A) HCT116 and (B) HT29 cells were treated with compound 3282-0486 at the indicated doses for 24 h. After incubation, the cells were fixed, stained with PI, and analysed using flow cytometry. The cell cycle distribution was analysed and quantified in the right bar graph. The experiment was performed in triplicate.

In advanced malignancies, TGF-β and TGFβR1 function a critical role in tumour progression.^34^ TGF-β and TGFβR1 promote invasion and metastasis by inducing EMT, remodelling the extracellular matrix, and suppressing antitumor immune responses^4^^,^^35^^,^^36^. Moreover, TGF-β participates in the DNA double-strand break repair pathway, especially during the S phase, thereby enhancing tumour cell survival^5^. Herein, the cell cycle distribution of colorectal cancer cells after TGFβR1 inhibitor 3282-0486 treatment was analysed and quantified (Figure 6). The percentages of G0/G1, S, and G2/M in untreated cancer cells were maintained at around 58%, 18%, and 23% in HCT116 cells, and 59%, 15%, and 25% in HT29 cells, respectively. After treating compound 3282-0486 at various concentrations, the cell numbers in G2/M increased to 24–46% in HCT116 and 25-48% in HT29 in a dose-dependent manner. At the concentration of 10 µM, the SubG0 cell population in HCT116 cells increased, suggesting the reduction of DNA content compared to G0/G1 phase cells, potentially indicating apoptosis or DNA fragmentation. These results demonstrate that the compound 3282-0486 modulated the cycle distribution of colorectal cancer cells, promoting G2/M accumulation and SubG0 phase increase.

### 3282-0486 Inhibited colorectal cancer cell migration

**Figure 7.**
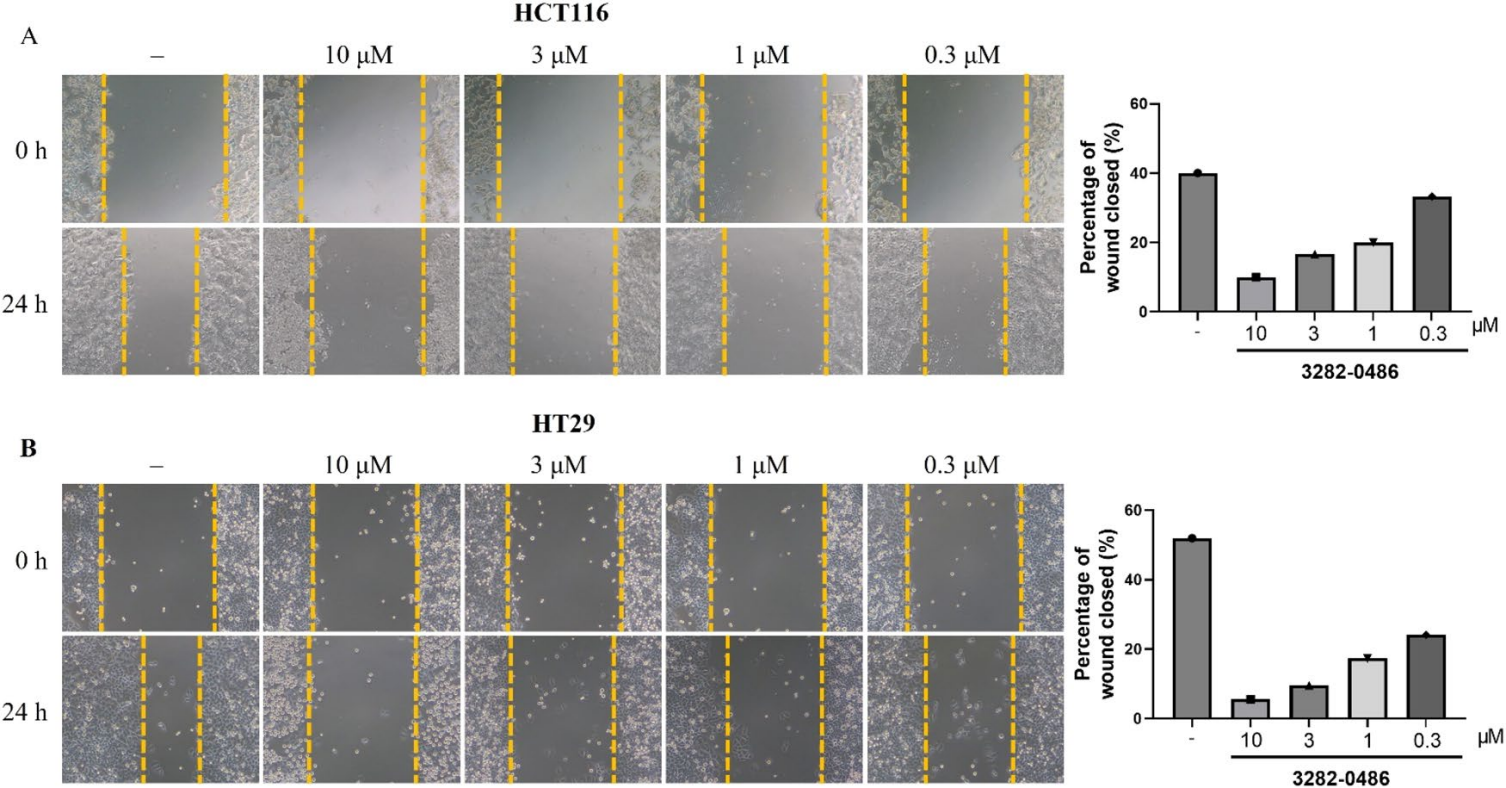
Effects of compound 3282-0486 on cell migration in colorectal cancers. (A) HCT116 and (B) HT29 were scratched with tips, treated with 10 ng/mL TGF-β and compound 3282-0486 at the indicated dose for 24 h. The distance of the wound was measured and quantified in the right bar graph.

TGF-β receptor and its downstream SMAD signalling are known to induce EMT and promote tumour migration. To evaluate the anti-migration effect of compound 3282-0486, scratch assays were performed. Colorectal cancer cells, HCT116 and HT29, were seeded in 24-well plates, scratched with a pipette tip, and imaged following compound treatment. Cell migration was assessed by comparing images taken at time zero and 24 h post-scratching (Figure 7). In untreated cells, wound closure reached 40% in HCT116 and 52% in HT29. Treatment with compound 3282-0486 led to a dose-dependent reduction in wound closure, with percentages decreasing to 5-10% at 10 μM, 10-17% at 3 μM, 17-20% at 1 μM, and 24-33% at 0.3 μM in both cell lines. These results indicated that compound 3282-0486 effectively inhibited colorectal cancer cell migration in a dose-dependent manner.

### Exploring compound selectivity across the kinome

Protein kinases regulate protein function by transferring a phosphate group from ATP to the hydroxyl group of amino acids. Despite the structural similarity, inactive kinase states vary significantly. Ideally, inhibitors should selectively bind specific kinases. However, achieving selective protein kinase inhibition is challenging, which emphasises the need for highly specific inhibitors^37^. TGFβR1 is classified as a member of the tyrosine kinase-like (TKL) serine/threonine kinases family^38^. The similarity of the ATP binding site among serine/threonine kinases limited the selectivity of inhibitors, potentially leading to off-target effects and consequently undesirable adverse effects. Herein, assessing the selectivity of compound 3282-0486 across a diverse range of kinases within the human kinome is essential. Compound 3282-0486 was assessed at a concentration of 1 μM against a panel of 60 kinases. The results indicated that compound 3282-0486 selectively inhibited TGFβR1 without significant inhibition of kinases from other families.

### 3282-0486 Reduced TGFβ downstream signalling and induced cell apoptosis

The canonical TGF-β signalling pathway mediated by Smad transcription factors is involved in TGF-β-mediated EMT, invasion, and proliferation of cancer cells. Phosphorylation of Smads via TGFβR1 and TGFβR2 and the translocation of p-Smad2/3 and bound Smad4 to the nucleus, regulates gene transcription of EMT protein expression.^39^ Hence, western blot analysis examined the downstream protein signalling of TGF-β to verify the effect of compound 3282-0486 in the inhibition of TGFβR1 (Figure 9). After treatment of compound 3282-0486 at 1 μM, the protein level of p-Smad2 decreased in a time-dependent manner in both HCT116 and HT29 colorectal cancer cells (Figure 9(A,B)). E-cadherin, a transmembrane protein that maintains cell adhesion, decreased after being treated with TGF-β, and increased after treatment with compound 3282-0486, also in a time-dependent manner in HCT116 (Figure 9(A)). Vimentin, as part of the cytoskeleton and intermediate filament, maintains cell structure and regulates cell shape and movement. Vimentin decreased 2 and 4 h after treatment, but rebounded at 8 and 24 h in HT29 (Figure 9(B)). The treatment of compound 3282-0486 for 48 h induced the cleavage of PARP1, which was primarily mediated by caspases and indicated cell apoptosis in both cell lines (Figure 9(C,D)). Together, these results suggested that the inhibition of TGFβR1 with compound 3282-0486 can effectively inhibit TGFβR1 downstream signalling and related EMT markers and also induce apoptosis.

## Discussion

In this study, novel small-molecule inhibitors of TGFβR1 were successfully identified using a structure-based virtual screening approach. Compound 3282–0487, containing a benzimidazole structure, was identified as a novel TGFβR1 inhibitor. Among its analogues, compound 3282-0486 emerged as the most potent inhibitor, inducing apoptosis and inhibiting migration in colorectal cancer cells. It modulated downstream signalling and showed high selectivity across the kinome. These findings position 3282-0486 as a promising therapeutic inhibitor targeting TGFβR1. Nowadays, SBVS is frequently used to identify bioactive compounds from large compound libraries. Using this approach, we identified 15 potential inhibitors. However, only two compounds showed more than 50% TGFβR1 inhibition at 10 μM (Table 1). The low hit rate in SBVS might be due to protein flexibility.^40^ Among the two compounds, compound 3282-0487 exhibited the better potency (IC_50_ 379.1 nM), prompting further analog testing. This led to the identification of additional submicromolar inhibitors, including 3282-0486 (IC_50_ 363.1 nM), 7491–0241 (IC_50_ 313.6 nM), and 7491-0127 (IC_50_ 391.7 nM).

Docking analyses revealed that the most potent compounds shared a benzimidazole scaffold, which formed critical hydrogen bonds with hinge residue H283 and additional interactions with I211 and V219. These interactions served as the anchoring interactions and were also complemented by hydrophobic contacts with residues, such as A230, K232, and L340 (Figure 2). Together, these interactions likely contributed to the compound potency. Benzimidazoles are organic compounds characterised by a benzene ring fused to an imidazole ring. The scaffold and its adjacent aromatic rings further enhanced binding. The benzimidazole-based scaffold has been identified as an effective structure for protein kinase inhibition. A distinct feature of benzimidazole scaffolds in kinase inhibition is their ability to engage the kinase hinge region through multiple binding modes. These interactions may occur via direct hydrogen bonding with the hinge backbone or through water-mediated bridges that stabilise inhibitor-hinge contacts. Such flexibility in hinge recognition is a notable advantage of benzimidazole scaffolds compared with many other kinase inhibitor classes^41–43^. Benzimidazoles are also relatively easy to synthesise and possess remarkable adaptability for structural modification. The benzimidazole-based compounds have been designed and developed for the inhibition of several tyrosine kinases with anti-cancer activity^42^^,^^43^. For instance, 4,5,6,7-tetrabromobenzotriazole (TBB) is an ATP site-directed inhibitor for protein kinase casein kinase-2 developed by Sarno *et al*; benzimidazole and benzoxazole derivatives by Potashman *et al* were developed for vascular endothelial growth factor-2 receptor (VEGFR-2); and 2-benzimidazolyl-9-(chroman-4-yl)-purinone derivatives were developed as Janus tyrosine kinase 3 (JAK3) inhibitors by Cole *et al*^44–46^ However, no studies to date have reported TGFβR1 inhibition by benzimidazole-based compounds. To the best of our knowledge, this study is the first to demonstrate that benzimidazole-based compounds can inhibit TGFβR1, revealing a previously unrecognised pharmacological potential of this chemical class.

**Figure 8.**
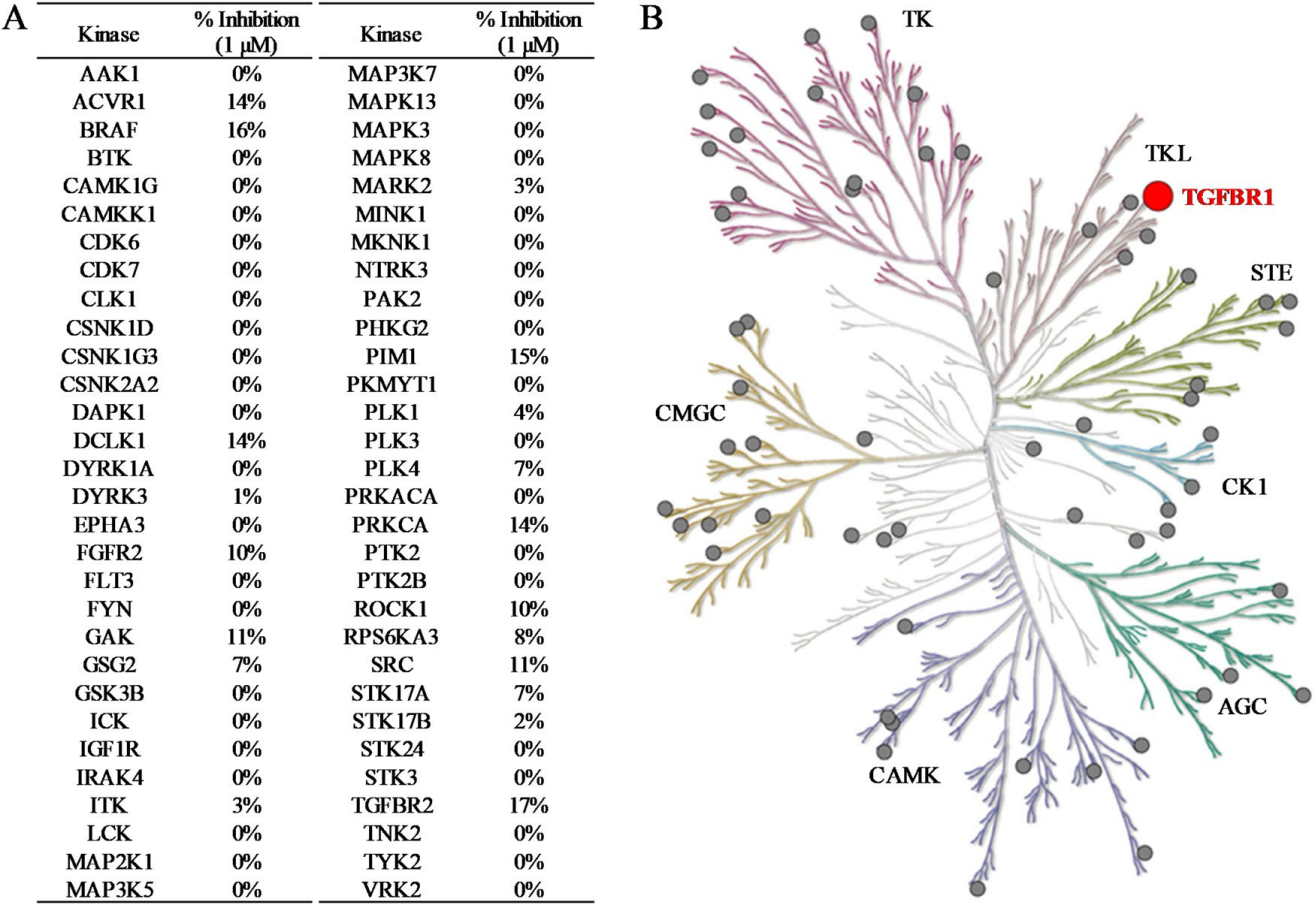
The kinase selectivity profile of compound 3282-0486**. (**A) Compound 3282-0486 was assessed across a panel of 60 kinases at 1 µM. (B) The inhibition percentage over 50% was marked as a red dot, and less than 50% was marked as a gray dot.

Importantly, the structural novelty of these inhibitors was confirmed through a Tanimoto similarity analysis against known TGFβR1 inhibitors from CHEMBL (Figure 4). The low similarity scores (< 0.208) and distinct clustering of compounds such as 3282-0487 and 8016-6700 underscore their uniqueness, suggesting that these scaffolds occupy a previously unexplored chemical space. The structural novelty may be particularly valuable in overcoming limitations of existing inhibitors, such as poor selectivity and toxicity. Moreover, it provides an opportunity to explore new avenues for therapeutic innovation. Although clinical use of benzimidazole-based kinase inhibitors remains limited, likely due to the poor selectivity and the broad tissue distribution of kinases, which heightens the risk of off-target effects^43^. Their activity and selectivity profiles are largely determined by the specific substitutions on the benzimidazole scaffold^42^. Herein, the compound selectivity across the kinome was performed. The kinase selectivity profiling confirmed that compound 3282–0486 selectively inhibited TGFβR1 without significant off-target activity across a panel of 60 kinases (Figure 8). Among the five tyrosine kinases selected for tests, only BRAF and TGFβR2 exhibited less than 20% inhibition. The compound showed promising selectivity and can be further optimised for therapeutic development.

**Figure 9.**
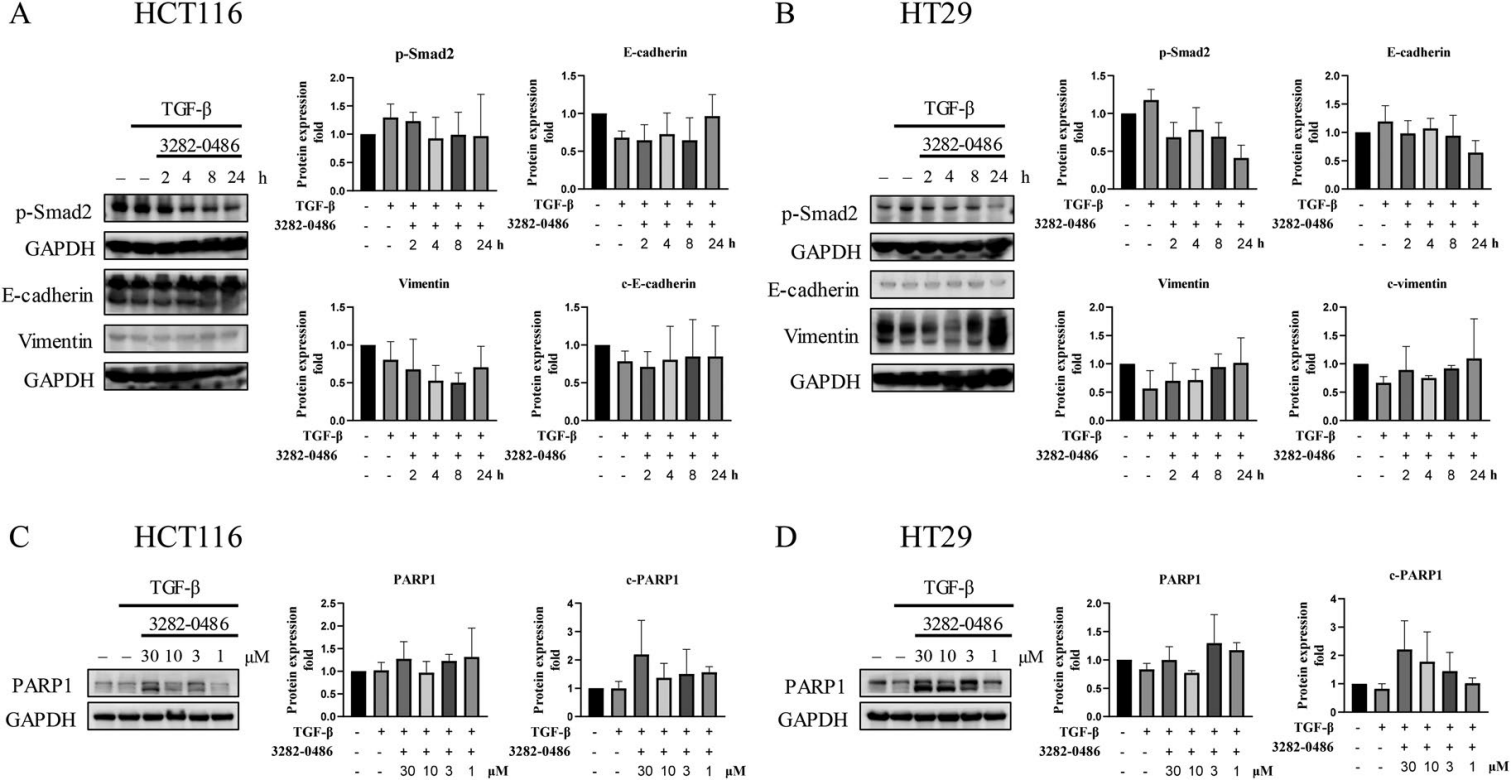
Effects of compound 3282-0486 on downstream signalling proteins in colorectal cancer cells. Cells were treated with TGF-β and compound 3282-0486 at the indicated dose and duration. The protein was collected and further assessed by Western blot analysis. (A, B) Cells were treated with 10 ng/mL TGF-β for 2 h and compound 3282-0486 at 1 μM for 2, 4, 8, and 24 h. (C, D) Cells were treated with 10 ng/mL TGF-β and compound 3282-0486 at 30, 10, 3, and 1 μM for 48 h. The experiment was performed in triplicate.

SAR analysis of 3282–0487 and its analogues provided deeper insight into the molecular determinants of activity. Electron-donating substitutions on Ring A modestly improved potency. For example, alkyl, alkoxy, and amino groups increase the electron density of Ring A and are promising substitution options. Replacing phenyl with thiophene or furan on Ring B helped avoid unfavourable hydrophobic regions and enhanced inhibition. This suggests that other five-membered heterocycles, for example, pyrrole, selenophene, imidazole, and isoxazole could further enhance inhibitory potency. The orientation and rigidity of the hydrophobic amide linker between the benzimidazole and Ring B were also critical, with optimal alignment to surrounding electrostatic features (PES and NESs) driving improved activity. In addition, we examined available protein–compound complex structures of TGFβR1 and observed that many inhibitors form hydrogen bonds with residues K232 or D351 (Figure S4), which is the aspartate in the DFG motif. Since these residues are located near Ring A, attaching polar atoms at this position may enable additional hydrogen bonds with K232 or D351 and improve potency. These findings offer a rational framework for future design and optimisation.

Cellular assays further validated the biological relevance of these compounds. Cytotoxicity testing in HCT116 and HT29 colorectal cancer cell lines revealed that compound 3282-0486 exhibited the most potent activity with IC_50_ values of 3.19 μM and 4.11 μM, respectively (Figure 5). HCT116 cells are characterised by wild-type P53, Ki-ras mutation, wild-type APC, and high aggressiveness. HT29 cells exhibit P53 mutation, wild-type Ki-ras, APC mutation, and intermediate capacity for differentiation. Further, HCT116 cells show a lower level of mismatch repair (MMR) genes and possess a microsatellite-unstable genotype. While HT29 cells exhibit a normal level of MMR genes, they have a microsatellite-stable genotype^47^^,^^48^. The growth inhibitory effects on two colorectal cancer cells with different characteristics assure that compound 3282-0486 could inhibit the proliferation of cancer cells, even gene expression variations.

The inhibitor identified in this work exhibited promising enzyme-based and cell-based activity; however, further in vivo studies are needed to evaluate its anti-tumour efficacy, pharmacokinetic properties, and potential toxicity. Although the compound inhibited TGFβR1 with an IC_50_ value of 363.1 nM, additional structural optimisation is needed to improve its efficacy against colorectal cancer cells. In addition, the current SAR analysis is mainly based on the available analogs in the ChemDiv collection, which restricts the range of chemical modifications that can be systematically explored. Expanding the series through the synthesis of additional derivatives with broader modifications at the Ring A and Ring B positions will be important for developing a more comprehensive understanding of the SAR. These considerations suggest that comprehensive SAR-guided optimisation, followed by in vivo validation, will be helpful in advancing this scaffold towards preclinical development.

## Conclusion

In this study, we used the SBVS strategy to identify novel TGFβR1 inhibitors from the ChemDiv library. Compound 3282–0487 and structurally related compounds were identified and evaluated for TGFβR1 inhibition. Four of these compounds showed potent TGFβR1 inhibition with IC_50_ values of 300–400 nM. The analyses of molecular docking and SAR revealed the key interactions between these inhibitors and the TGFβR1 binding site. Compound 3282–0486 exhibited anti-proliferative activity against colorectal cancer cells, inducing cancer cell apoptosis and DNA fragmentation. Additionally, compound 3282–0486 dose-dependently reduced cancer cell migration. Further evidence of its effectiveness in inhibiting proliferation and migration was provided by changes in downstream TGFβR1 signalling proteins, including p-Smad2, EMT markers, and the cleavage of PARP1. Importantly, compound 3282–0486 also demonstrated selectivity for TGFβR1 across the human kinome. Overall, these findings establish compound 3282–0486 as a novel and promising TGFβR1 inhibitor with significant potential in colorectal cancer treatment.

## Supplementary Material

Supporting_information_2025Nov_ Clean.docx

## Data Availability

The datasets used and/or analysed during the current study are available from the corresponding author on reasonable request.
