# Supplementary material for: Identification and biological evaluation of benzimidazole-based compounds as novel TGFβR1 inhibitors
Source: J Enzyme Inhib Med Chem. 2026 Jan 21;41(1):2600746. doi: 10.1080/14756366.2025.2600746 (PMC12829415; doi:10.1080/14756366.2025.2600746)
Supplement: Supporting_information_2025Nov_ Clean.docx [file IENZ_A_2600746_SM0009.docx]

**Supplementary Figures**

**Identification and Biological Evaluation of Benzimidazole-Based Compounds as Novel TGFβR1 Inhibitors**

**Supplementary figure 1.** **Activity cliffs and SAR analysis of selected compounds.** (A) Electrostatic and hydrophobic pocket regions were mapped based on compound 7491-0241. The surrounding pocket features (B) negative electrostatic sites (blue). (C) The lower pocket harbors an unfavorable hydrophobic site (Pink). The rear pocket contains (D) a positive electrostatic site (red).

**Supplementary figure 2. Dose-response curves of hit compounds.** Four hit compounds with TGFβR1 inhibitory activity > 50% were tested for dose-response curves.

**Supplementary figure 3. Triplicate experimental data of effect of compound 3282-0486 on down-signaling proteins in colorectal cancer.** Cells were treated with TGF-β and compound 3282-0486 at the indicated dose and duration. The protein was collected and further assessed by Western blot analysis. (A, B) Cells were treated with 10 ng/mL TGF-β for 2 h and compound 3282-0486 at 1 μM for 2, 4, 8, and 24 h. (C, D) Cells were treated with 10 ng/mL TGF-β and compound 3282-0486 at 30, 10, 3, and 1 μM for 48 h.

**Supplementary figure 4. Binding conformations of TGFβR1 inhibitors complexed in crystal structures.** In these structures, the inhibitors form hydrogen bonds with residues K232 or D351. The TGFβR1 structure is shown as a cartoon, and the binding site is displayed as sticks. Hydrogen bonds are indicated by dashed green lines.
